# Differences in plasmatic and CSF Alzheimer's Disease biomarkers considering ApoE genotype in mild cognitive impairment and subjective cognitive decline

**DOI:** 10.1002/alz70856_104334

**Published:** 2025-12-26

**Authors:** Beatriz Ramos Lucente, Isadora Cristina Ribeiro, Luis E. Santos, Ananssa Silva, Thaís Lopes Pinheiro, Liara Rizzi, Ítalo Karmann Aventurato, Brenda Costa Gonçalves, Marjorie Cristina Rocha da SIlva, Gustavo Bruniera Peres Fernandes, Fernanda G. De Felice, Marcio Luiz Figueredo Balthazar

**Affiliations:** ^1^ UNICAMP, Jundiaí, São Paulo, Brazil; ^2^ Universidade Estadual de Campinas (UNICAMP), Campinas, SP, Brazil; ^3^ D'Or Institute for Research and Education, Rio de Janeiro, RJ, Brazil; ^4^ UNICAMP ‐ UNIVERSIDADE ESTADUAL DE CAMPINAS, CAMPINAS, SP, Brazil; ^5^ Hospital Israelita Albert Eisntein, São Paulo, São Paulo, Brazil; ^6^ D’Or Institute for Research and Education, Rio de Janeiro, Brazil; ^7^ University of Campinas (Unicamp), Campinas, SP, Brazil

## Abstract

**Background:**

Alzheimer's disease (AD) is the most prevalent neurodegenerative disorder, and ApoE ε4 allele is the most relevant genetic risk factor for sporadic AD. Although it is well‐known that ApoE ε4 is related to brain amyloidosis, much less is known whether this genetic risk factor may influence Aβ and tau plasmatic levels in the mildest clinical phase: mild cognitive impairment (MCI) and subjective cognitive decline (SCD). We evaluated if plasmatic (Aβ40, Aβ42, *p*‐tau181 e t‐tau) and cerebrospinal fluid (CSF) (Aβ42, ptau‐181, ptau‐231 and t‐tau) biomarkers differed in patients considering different APOE genotypes (ε2ε3, ε3ε3 and ε3ε4).

**Method:**

64 older adults (MCI: 36, SCD: 12, AD: 3, control: 13), mean age: 66± 6.6 years old, underwent blood and CSF tests. Roche's Elecsys electrochemiluminescence immunoassay measured the quantification of CSF t‐Tau, *p*‐Tau 181, and Aβ42 proteins. Plasma biomarkers Aβ42, Aβ40, *p*‐Tau 181, and t‐Tau were measured on an automated SIMOA HD‐X immunoassay equipment. Real‐time PCR amplification was performed using the 7500 System software (Thermo Fisher Scientific). The APOE genotype determination was based on analyzing single nucleotide polymorphisms rs429358 and rs7412. ANOVA evaluated differences between groups with Bonferroni's post hoc test. Significance was set at *p* < 0.05.

**Result:**

We found that the ApoE ε3ε4 group had a lower concentration of CSF Aβ42 than the ε2ε3, *p* = 0.02. No differences were found in plasmatic levels of any AD biomarkers.

**Conclusion:**

Plasmatic AD biomarkers did not differ regarding APOE genotype in our sample, but we find a difference in CSF biomarker and the APOE genotype. So, our findings confirm the influence of APOE ε4 allele in central nervous system amyloid levels.

